# MMHub, a database for the mulberry metabolome

**DOI:** 10.1093/database/baaa011

**Published:** 2020-03-11

**Authors:** Dong Li, Bi Ma, Xiaofei Xu, Guo Chen, Tian Li, Ningjia He

**Affiliations:** 1 State Key Laboratory of Silkworm Genome Biology, Southwest University, No. 2, Tiansheng Road, Beibei, Chongqing 400715, China; 2 College of Computer and Information Science, Southwest University, No. 2, Tiansheng Road, Beibei, Chongqing 400715, China

## Abstract

Mulberry is an important economic crop plant and traditional medicine. It contains a huge array of bioactive metabolites such as flavonoids, amino acids, alkaloids and vitamins. Consequently, mulberry has received increasing attention in recent years. MMHub (version 1.0) is the first open public repository of mass spectra of small chemical compounds (<1000 Da) in mulberry leaves. The database contains 936 electrospray ionization tandem mass spectrometry (ESI-MS^2^) data and lists the specific distribution of compounds in 91 mulberry resources with two biological duplicates. ESI-MS^2^ data were obtained under non-standardized and independent experimental conditions. In total, 124 metabolites were identified or tentatively annotated and details of 90 metabolites with associated chemical structures have been deposited in the database. Supporting information such as PubChem compound information, molecular formula and metabolite classification are also provided in the MS^2^ spectral tag library. The MMHub provides important and comprehensive metabolome data for scientists working with mulberry. This information will be useful for the screening of quality resources and specific metabolites of mulberry.

**Database URL:** https://biodb.swu.edu.cn/mmdb/

## Introduction

Mulberry (*Morus* spp.) is widely recognized as an important economic crop plant and is irreplaceable in the sericulture industry. Recently, increasing attention has been paid to mulberry bioactive metabolites, which have a variety of pharmacological benefits (1–4). These metabolites include 1-deoxynojirimycin (DNJ) (5), flavonoids (6, 7), polysaccharides (8) and anthocyanins (9). Any single plant species can produce a huge array of metabolites, with estimates ranging from 5000 to the tens of thousands (10, 11). Mulberry is also considered as a traditional medicine that is rich in bioactive metabolites.

Over the past 10 years, metabolomics based on compound separation and mass spectrometry has evolved from a little-known branch of analytical chemistry to a mainstream enterprise. It is now possible to identify and quantify hundreds of metabolites in a single analysis (12–15). A widely targeted metabolic profiling method (13) has been successfully applied to the studies of rice, maize, qingke and barley metabolomes, providing MS^2^ spectral tag (MS2T) libraries with 840, 983 and 2059 distinct molecular features, respectively. However, these valuable mass spectral data and research products should be presented in a more visual and accessible way than simply listing supporting data with mass-to-charge ratio (*m/z*) and major peaks in a digital format. Tandem mass spectrum (MS/MS) spectral data are important experimental data that support life science research (16). Although thousands of metabolites with distinct chemical structures and mass spectra are listed in published mass spectral databases like MassBank (17), HMDB (12) and METLIN (18), it is impossible for researchers to screen for metabolites only detected in a particular species from these knowledgebases.

The draft genome sequence of *Morus notabilis* has been reported (19), and a supporting platform (MorusDB) is available for researchers to search and analyze the mulberry genomics and related data (20). However, at present, there is no comprehensive database of information about the composition, distribution and structures of mulberry metabolites. By combining a widely targeted metabolic profiling approach and high-throughput quantitative analysis, we created MMHub (version 1.0), a publicly available mulberry metabolome database of small chemical compounds (<1000 Da). This database is now available for other researchers to search and analyze the distinct mass spectra, distribution and structures of mulberry metabolites.

## Materials and Methods

### Construction and MMHub

MMHub (version 1.0) was constructed using LAMP (CentOS Linux release 7.4.1708, Apache 2.4.6, MariaDB 5.5.56 and PHP 5.4.16), which is an efficient platform for developing a comprehensive database. All metabolome data and information were stored in a MySQL relational table, which allowed for structural data, spectra data and other useful data about metabolites to be entered manually and indexed as quickly as possible.

### Plant materials

Mulberry (*Morus* spp.) resources were cultivated at the Mulberry Germplasm Nursery in Southwest University, Chongqing, China. Leaves collected from the mulberry plants were used for MS2T library construction. A total of 91 resources with two replicates were sampled in June 2018. Two or three mature leaves were collected from the longest branch of each resource and frozen in liquid nitrogen. All 91 resources (Table S3) were classified into three species according to Zeng *et al.*’s study (21): 89 *Morus alba* L., one *Morus nigra* and one *M. notabilis*. Samples were then freeze-dried, ground and extracted (22). The sample extracts were analyzed using a liquid chromatography electrospray ionization tandem mass spectrometry system in positive ion mode (Ultra High-performance liquid chromatography, Thermo Scientific™ Dionex™ UltiMate™ 3000; MS, Q Exactive hybrid quadrupole-Orbitrap mass spectrometer; Thermo Fisher Scientific, Waltham, MA, USA).

**Figure 1:**
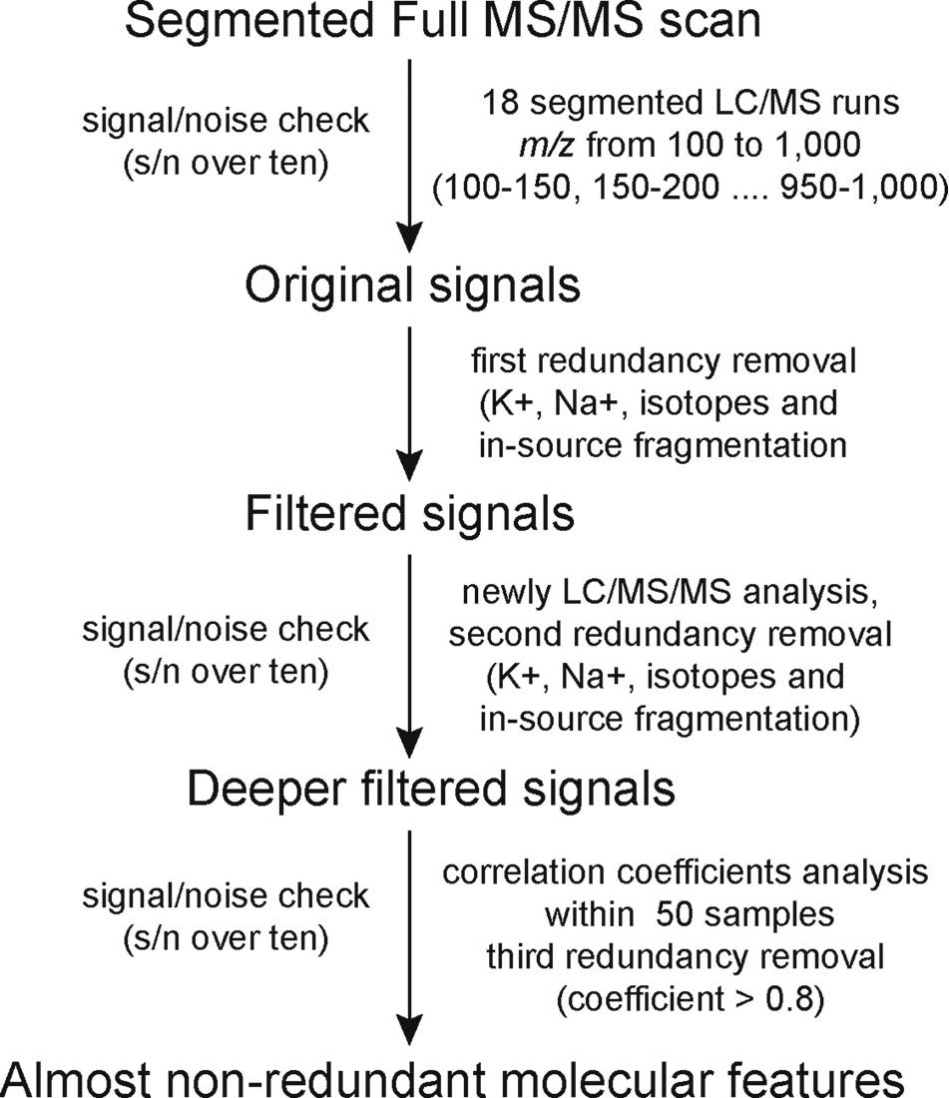
The workflow of MS2T library construction based on HPLC-MS/MS and its practical application in mulberry.

**Figure 2:**
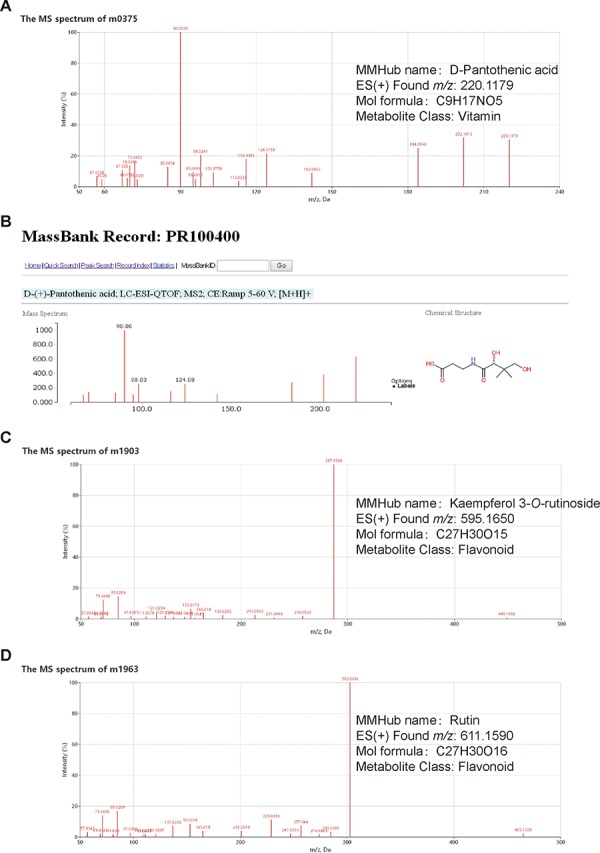
Examples of metabolite identification based on database comparisons and specific fragmentation patterns in MMHub. Molecular feature m0375 (**A**) was identified as D-pantothenic acid by comparison with MassBank (**B**). Two molecular features (m1903 and m1963) were annotated as kaempferol 3-*O*-rutinoside (**C**) and rutin (**D**) based on flavonoid-specific fragmentation patterns. Identities of three metabolites shown in this figure were confirmed by comparison with authentic standards.

### MS2T library construction

A segmented full MS/MS scan strategy developed from the widely targeted metabolomics approach was used to construct the mulberry MS2T library (Figure 1). This strategy comprised 18 full MS/MS runs (segmented with 50 *m/z*) instead of thousands of multiple ion monitoring-enhanced product ions transitions in 113 runs (22). Original chromatographic peaks (signals) and related mass spectra were manually checked. Further filtering was conducted to remove potentially redundant signals including those of isotopes, in-source fragmentation products, K^+^, Na^+^ and NH4^+^ adducts and dimerzations. The signal/noise (s/n) check and three rounds of redundancy removal were performed using the same standards with the software that we developed in-house. Supporting information such as PubChem compound information, molecular formula and main fragments were also added to the MS2T library according to Fernie’s recommendation (23).

### Metabolite identification and annotation

Metabolite identification/annotation was based on the accurate *m/z*, retention time (RT) and fragmentation patterns. The accuracy of metabolite identification (from high to low) was divided into four levels (A–D). Level A was the most accurate identification, indicating that those metabolites had the same RT (± 0.1 min) and mass spectra as those of authentic standards. Level B indicated those metabolites showed >85% match rate when their main fragments were searched against public databases (MassBank, KNApSAcK, HMDB and METLIN), or showed specific fragmentation patterns. Metabolites only with confident *m/z* (|error|≤10 ppm) by comparison with references or detected in other species were defined as level C and D (relatively low accuracy). The proportions of metabolites in categories C and D were relatively low.

### Quantification of metabolites

To improve the sensitivity and accuracy of quantification, 936 molecular features were analyzed in two *m/z* ranges (from 100 to 400 and from 400 to 1000). The quantitative calculations were conducted using Thermo Scientific™ Xcalibur™ software v. 2.2. Each metabolite was quantified with accurate mass tolerance (units, 20 ppm) and precision (decimals, 0.0001). To verify data stability and reproducibility, a mixture of 50 randomly chosen extracts was repeatedly analyzed (*n* = 6) as the reference control through the analytical procedure. Each set of 20 samples and the relative content of each metabolite were normalized to the average in the reference control. The relative content of each metabolite in 91 mulberry resources has been deposited in the MMHub as its log^2^ value.

**Figure 3:**
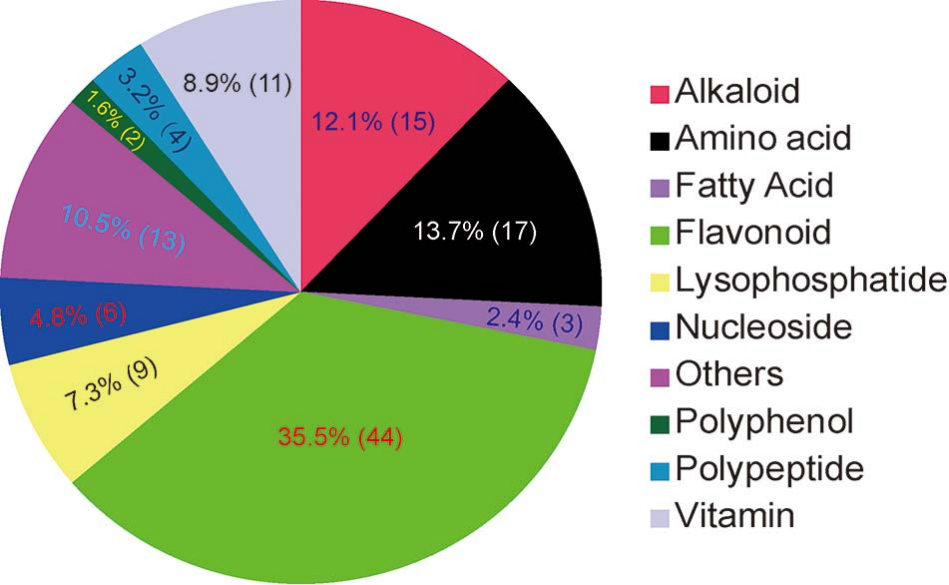
Classification of 124 identified/annotated metabolites in MMHub. The numbers of metabolites in each classification are shown in parentheses.

**Figure 4:**
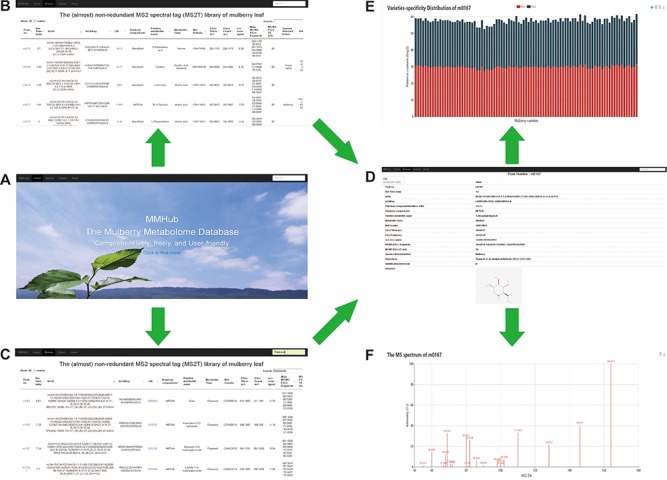
Snapshots of searches for metabolites and related information in MMHub. Researchers can retrieve general information about metabolites by basic browsing (**A** and **B**) or specific searches (**C**). Detailed information including available compound information (**D**), quantitative data (**E**) and mass spectrum (**F**) retrieved by clicking the ‘Peak no.’ link.

## Results

### Database description

We analyzed the metabolome of mulberry using a widely targeted metabolomics approach based on high performance liquid chromatography-tandem mass spectrometry (HPLC-MS/MS) (13). By applying this segmented full MS/MS scan strategy (Figure 1), metabolites with mass range from *m/z* 100 to 1000 were detected with high sensitivity in 18 LC-MS/MS runs. In total, 4924 chromatographic peaks (signals) with s/n > 10 were manually checked. To produce a matrix with fewer biased and redundant data, redundancies caused by signals from isotopes, in-source fragmentation products, K^+^, Na^+^ and NH4^+^ adducts and dimerzations were removed using in-house software written in Perl (22). After the first redundancy removal, 2319 signals were obtained. To produce optimal mass spectra data and remove potential redundancies, a high-concentration mixture of compounds was subjected to LC-MS/MS analysis under the data-dependent MS^2^ (dd-MS^2^) mode. These new data were run through redundancy removal software (second redundancy removal) and generated a data matrix with 1577 signals.

We expected that transitions derived from the same metabolites would be strongly correlated among different samples. Thus, a pre-test quantitative analysis of 50 mulberry resources was conducted and correlation coefficients between transition pairs were calculated. We found that 641 metabolites with high correlation coefficients (>0.9 for metabolites eluted earlier than 2.5 min or > 0.8 for metabolites eluted later than 2.5 min). The same retention time (±0.1 min) were considered as another criterion of redundancy (third redundancy removal). Finally, an MS2T library containing 936 molecular features with almost no redundancy was obtained and is reported as recommended previously (Tables S1 and S2) (23).

**Figure 5:**
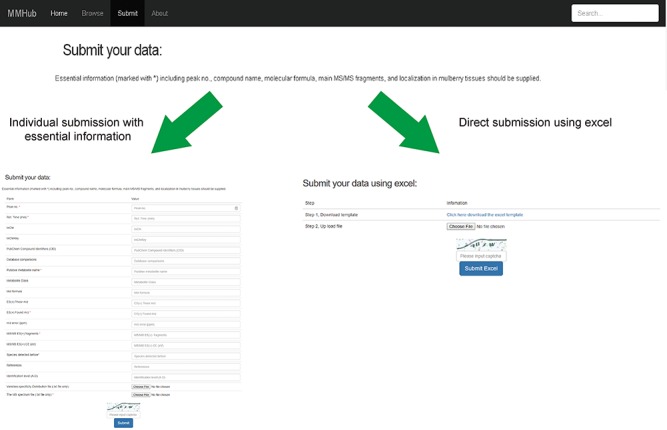
User guide for uploading and submission of mass spectra data into MMHub.

### Identification and annotation of metabolites in mulberry leaves

In MMHub (version 1.0), 37 commercially available standards were identified using the same profiling conditions as those used to analyze the extracts. Eighty-seven metabolites were putatively identified by querying MS/MS spectra data against the literature or databases (MassBank, KNApSAcK, HMDB and METLIN) (Figure 2A and B). A number of metabolites were identified by analyzing specific fragmentation patterns. For example, two flavonoids, m1903 (RT 7.35 min, *m/z* 595.1650, error − 1.18 ppm) and m1963 (RT 6.81 min, *m/z* 611.1590, error − 2.78 ppm) were identified as follows: the diagnostic fragment ions of kaempferol were *m/z* 287.0546, 153.0179 and 121.0284; the natural loss of hexoside (162 Da, 449.1068 → 287.0546) and rhamnoside (146 Da, 595.1650 → 449.1068) moieties were revealed in the mass spectrum (Figure 2A); the diagnostic fragment ions of quercetin were *m/z* 303.0494, 257.0440, 229.0491 and 153.0180; and the natural losses of the hexoside and rhamnoside moieties were revealed in the mass spectrum (Figure 2B). Subsequent comparative analysis of standards confirmed that m1903 and m1963 were kaempferol 3-*O*-rutinoside and rutin (quercetin 3-*O*-rutinoside), respectively. Their glycosylation sites were determined and were consistent with our predictions. MMHub contains 124 identified or tentatively annotated metabolites and 90 metabolites with associated chemical structures including 44 flavonoids, 15 alkaloids, 17 amino acids, 9 lysophosphatides, 11 vitamins, 4 polypeptides and several other kinds of metabolites (Figure 3).

### User interface

To provide a user-friendly way to access all metabolomics data mentioned above, we constructed MMHub (version 1.0), which allows researchers to browse and search the data efficiently. The MMHub database is navigated by a top menu, with four major sections: Home, Browse, About and a powerful string search box (Figure 4A). We have not provided any user guidelines because it is extremely easy for users to find information. For example, by clicking on the ‘Click to find more’ hyperlink on the MMHub homepage, a new page opens with detailed information about metabolomics (Figure 4B).

A powerful string search box is located at the right top of each page in the MMHub database. Any string can be submitted as a query to retrieve corresponding records from the database. By searching for ‘flavonoid’, for example, matching hits with high scores are listed in tabular format (Figure 4C). MMHub is fully searchable with scrips to view and extract metabolomics information, including peak no., RT, structure identifiers, international chemical identifier keys, PubChem compound identifiers, metabolite name, metabolite class, molecular formula and other information supported in the MS2T library (Tables S1 and S2). Clicking on the link ‘Peak no.’ in the browsing page takes the reader to a page with detailed information about that peak, including basic qualitative data (RT, *m/z*, main fragments and available compound information) (Figure 4D), quantitative data (specific distribution in 91 mulberry resources with two biological duplicates) (Figure 3E) and original mass spectrum (Figure 4F). A key feature of MMHub is that it provides links for known compounds to the PubChem database, where more features are listed (2D and 3D structures).

As an open metabolome database for mulberry, we expect to accommodate more metabolite data for other mulberry tissues, which can be added by researchers from any institution. By clicking on the ‘Submit’ hyperlink, any user can submit metabolite data to MMHub (Figure 5). Essential information (marked with *) including peak no., compound name, molecular formula, main MS/MS fragments and localization in mulberry tissues should be supplied. Users can choose to submit data manually one by one or directly upload data in file format. To ensure the authenticity and reliability of the data, all uploaded data will be temporarily stored in our local server before being deposited in MMHub. New supporting data that have been contrasted and filtered against available data in MMHub will be updated regularly and displayed to all users.

## Discussion

Mulberry has been identified as a potential functional nutraceutical food in recent years (24). Bioactive compounds in mulberry have been shown to prevent and treat hyperglycemia (25, 26), hyperlipidemia (3, 27), Alzheimer’s disease (28) and cancer (29). Mass spectral data are important experimental data for research on bioactive compounds. MMHub (version 1.0) is the first public repository of mass spectra of small chemical compounds (<1000 Da) in mulberry leaves. Although our ESI-MS^2^ data were obtained under non-standardized and independent experimental conditions, Volna *et al.* found that the fragmentation patterns are almost identical for all tandem mass analyzers and that only the ratios of the product ions differ somewhat (16, 30). For instance, m0167 and m0375 are identified as DNJ and D-pantothenic acid, respectively, in MMHub and show similar fragmentation patterns and main fragments in the HMDB (HMDB ID: HMDB0035359) and MassBank (MassBank record: PR100400) databases (Figure 2B). Actually, 87.1% of 124 identified metabolites (108/124) in MMHub are also listed in other public databases (MassBank, METLIN, HMDB and KNApSAcK).

The widely targeted metabolic profiling method based on HPLC-MS/MS is not limited to a single mulberry species or particular tissue (Figure 1). This approach has been used to construct multiple MS2T libraries including 983 molecular features for maize kernels (22), 840 for rice leaves (31) and 2059 for qingke and barley (14). Primary and secondary metabolites like amino acids, nucleic acids, vitamins, flavonoids and vitamins can be efficiently detected using this high-throughput method. Qualitative and quantitative variations in metabolism are regarded as the ‘metabotype’ (32), which represents the bridge between the genotype and the phenotype of a plant (11). Recently, metabolomics combined with broad profiling approaches like genomics and transcriptomics has become an essential method to explore the diversity of plant metabolism and its underlying molecular mechanisms (22, 33, 34). This comprehensive metabolome database will provide a starting point for research on bioactive metabolites and will be useful for the identification of new candidate genes in mulberry.

The existing public metabolome databases are richly annotated and can meet many of the needs of biochemists, clinical chemists, physicians, medical geneticists, nutritionists and members of the metabolomics community (12). MMHub is a specialized database for mulberry research. Although many metabolites remain unidentified, MMHub provided a comprehensive collection of data for research on the mulberry-specific metabolome. The database also includes related quantitative data for 91 mulberry resources with two biological duplicates. This information may be useful for mulberry breeders and plant biochemists to screen for quality resources and specific metabolites.

MMHub was constructed to help researchers mine for metabolomics information easily and effectively. It has several features and advantages: (i) all metabolomics information, including metabolites, structure, mass spectra and validated metabolite concentrations, are strictly curated and verified; (ii) as an initial metabolomics data repository for mulberry, it can be used to provide basic data for other databases; and (iii) MMHub is a work in progress. Newly identified metabolomics data for mulberry will be integrated into MMHub as quickly as possible, and submission queries from all researchers will be encouraged. The comprehensive metabolomic studies of multiple mulberry tissue including fruit and bark are carrying out. It is particularly exciting given that the tissue-specific metabolites, such as anthocyanidins, will greatly enrich the diversity of mulberry metabolites in MMHub. In addition, the agronomic traits of mulberry resources including leaf type, fruit size and fruit color will be integrated into the next version of MMHub.

## Conclusion

In summary, MMHub is a user-friendly, freely available and comprehensive metabolomics database that brings together data for thousands of endogenous mulberry metabolites. We believe that MMHub is unique. Although MMHub is a work in progress, our aim is to make it a special metabolomics repository for mulberry species. We are committed to continuously updating this database with strictly curated data, including more metabolites and more tissues. We expect that MMHub will be useful for applications in metabolomics, traditional medicines, biomarker discovery and general education.

## Availability

All metabolomics data deposited in this database are freely available to any researcher without restrictions.

Database name: MMHub.

Database URL: https://biodb.swu.edu.cn/mmdb/

*Conflict of interest*. The authors declare no competing financial interest.

## Supplementary Material

Table_S1-3_baaa011
